# Discovery of Galangin as a Potential DPP-4 Inhibitor That Improves Insulin-Stimulated Skeletal Muscle Glucose Uptake: A Combinational Therapy for Diabetes

**DOI:** 10.3390/ijms20051228

**Published:** 2019-03-11

**Authors:** Poonam Kalhotra, Veera C. S. R. Chittepu, Guillermo Osorio-Revilla, Tzayhri Gallardo-Velázquez

**Affiliations:** 1 Departamento de Biofísica, Escuela Nacional de Ciencias Biológicas, Instituto Politécnico Nacional, Prolongación de Carpio y Plan de Ayala S/N, Col. Santo Tomás, C.P. 11340 Ciudad de México, Mexico; kalhotrapoonam@gmail.com; 2 Departamento de Ingeniería Bioquímica, Escuela Nacional de Ciencias Biológicas, Instituto Politécnico Nacional, Av. Wilfrido Massieu S/N, Col. Unidad Profesional Adolfo López Mateos, Zacatenco, CP. 07738 Ciudad de México, México; veerareddy9@gmail.com (V.C.S.R.C.); osorgi@gmail.com (G.O.-R.)

**Keywords:** diabetes mellitus, molecular docking, galangin, DPP-4 inhibitor, combination therapy, skeletal muscle cell health

## Abstract

Dipeptidyl peptidase-4 (DPP-4) is a well-known therapeutic drug target proven to reduce blood glucose levels in diabetes mellitus, and clinically, DPP-4 inhibitors are used in combination with other anti-diabetic agents. However, side effects and skeletal muscle health are not considered in the treatment for diabetic patients. Recently, natural compounds have been proven to inhibit DPP-4 with fewer side effects. In this work, initially, molecular docking simulations revealed that a natural compound, Galangin, possess a binding energy of −24 KJ/mol and interaction residues SER 630 and TYR 547, that are responsible for potent DPP-4 inhibition. In vitro studies showed that galangin not only inhibits DPP-4 in a concentration-dependent manner but also regulates glucose levels, enabling the proliferation of rat L6 skeletal muscle cells. The combination of galangin with insulin benefits regulation of glucose levels significantly in comparison to galangin alone (*p* < 0.05). These findings suggest the beneficial effect of the use of galangin, both alone or in combination with insulin, to reduce glucose levels and improve skeletal muscle health in diabetes mellitus.

## 1. Introduction

Diabetes mellitus, a global disease characterized as an impairment in insulin secretion (type 1 diabetes mellitus), insulin resistance (type 2 diabetes mellitus) leads to hyperglycemia, and are two of the most important health problems faced by every country. The World Health Organization revealed that the prevalence of diabetes is about to reach 642 million by 2040 [1,2]. Insulin therapy is most commonly recommended for type 1 diabetes mellitus patients [3] whereas, sulfonylureas (e.g., glimepiride), meglitinides (e.g., repaglinide), biguanides (e.g., metformin), glitazones (e.g., rosiglitazone), α-glucoside inhibitors (acarbose), dipeptidyl peptidase-4 (DPP-4) inhibitors (e.g., sitagliptin) and combination therapies (e.g., metformin and rosiglitazone, insulin and metformin) are the most recommended therapies for type 2 diabetes mellitus patients, and all these antidiabetic therapies possess side effects like respiratory infection, hypoglycemia, hepatic and cardiovascular complications [4,5]. Recently, clinical trials were carried out to demonstrate the benefits of combination therapy in treating insulin resistance. Depending on the patient’s clinical characteristic, the second agent with a different mechanism of action, along with metformin, is recommended as dual combination therapy. Some of the dual combination therapies in clinical trials are sulfonylureas and insulin; metformin and insulin; vildagliptin and insulin; vildagliptin and metformin; an SLGT2 inhibitor and metformin [6,7,8], until today, dual combination therapies have not focused on improving skeletal muscle health.

Skeletal muscle accounts for 40–50% of our body mass and is the most crucial target organ responsible for insulin-dependent glucose uptake [9]. Therapeutic formulations favoring skeletal muscle health—especially in case of diabetes mellitus—is necessary because, in the insulin-resistant mechanism, there is impairment in insulin signaling pathways causing a lethal effect on glucose levels, resulting in abnormal glucose metabolism, insulin resistance, and low skeletal muscle mass [9,10]. Recently, clinical studies revealed that exercise led to reduced DPP-4 levels in blood and resulted in the improvement of insulin sensitivity [11]. DPP-4 inhibitors play a role in improving skeletal muscle cell health, insulin sensitivity and regulate glucose homeostasis.

**Figure 1:**
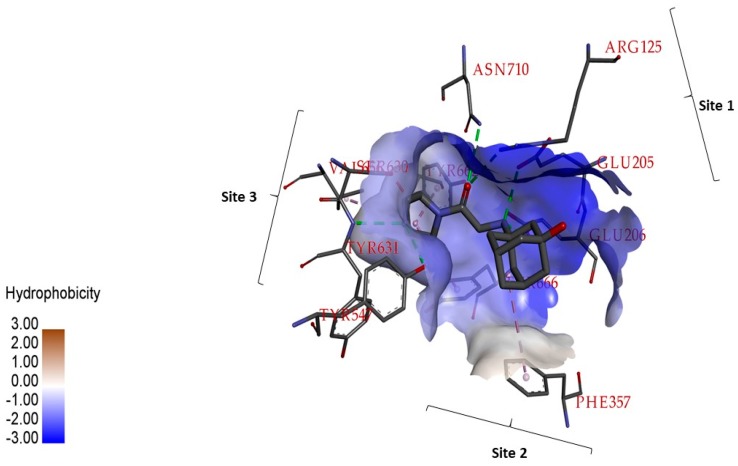
The important residues responsible for dipeptidyl peptidase-4 (DPP-4) inhibition, hydrophobic surface feature active site of DPP-4. Vildagliptin binding DPP-4 structure was retrieved from Protein Data Bank whose PDB ID is 6B1E, and the interactions are visualized using Discovery Studio tool.

Dipeptidyl peptidase-4 (DPP-4) is a serine protease; a glycoprotein that generally cleaves N-terminal dipeptides whose substrates are cytokines, growth factors, and incretin hormones usually expressed in different cells [12]. In humans and animals, upon ingestion of meals, intestinal L-cells release the incretin hormone to regulate glucose homeostasis [13]. In diabetic patients, incretin hormones are markedly reduced, because of serine protease DPP-4 [14]. The druggable region of DPP-4 includes: A catalytic triad with the interacting residues Ser630, Asp708 and His740; residues responsible for saline bridge Glu205, Glu206, and Tyr662; residues forming oxyanion activity Tyr47 and Ser 631; residues in the pocket Site S1 and S2 Arg125, Ser209, Phe357, Arg358, Tyr547, Ser631, Val656, Trp659, Tyr 62, Tyr666, Asn710, and Val711, as shown in Figure 1 [15,16,17,18,19]. DPP-4 inhibition by the chemicals sitagliptin and vildagliptin are proven to prevent degradation of incretin and are in clinical use [20]. These chemicals possess side effects like pancreatitis [21], gastrointestinal problems, angioedema, and infectious diseases. Hence, there is a necessity to identify new compounds to inhibit DPP-4 with fewer side effects.

Natural products are rich in chemical diversity and been proven to be exceptional in drug discovery, drug development and in therapeutic medicine [22,23,24]. The absence of acute toxicity and fewer side effects are the advantages associated with natural compounds. Natural products from food-derived bioactive compounds, including polyphenols, alkaloids, and peptides, are proven to reduce blood glucose levels in clinical trials [25]. Polyphenols are rich in food and have been proven to be antidiabetic [26], antitumor [27], antiviral [28,29], and anti-cardiovascular [30]. Recently, studies on metabolic syndrome patients revealed that diet plays a crucial role in plasma DPP-4 activity and as well, in incretin hormone activity [31]. Our earlier study had revealed molecular insights into the polyphenols inhibiting DPP-4 [32]. In the present study, in silico methods like molecular docking simulations were used to discover the ability of the natural flavonoid galangin (3,5,7-trihydroxyflavone) to bind at the active site of DPP-4 and experimentally validate inhibitory activity. In addition, the acute toxicity behavior of galangin on insulin-sensitive skeletal muscle cell was studied to demonstrate the proliferative behavior and glucose metabolism. Also, the role of galangin in combination with insulin was evaluated for the proliferative effect and glucose metabolism, in the case of the skeletal muscle system, to reveal the potential of combination therapy to treat diabetes mellitus. 

## 2. Results and Discussion

### 2.1. In Silico Studies on DPP-4 Enzyme Interactions with Flavanol Galangin

**Figure 2:**
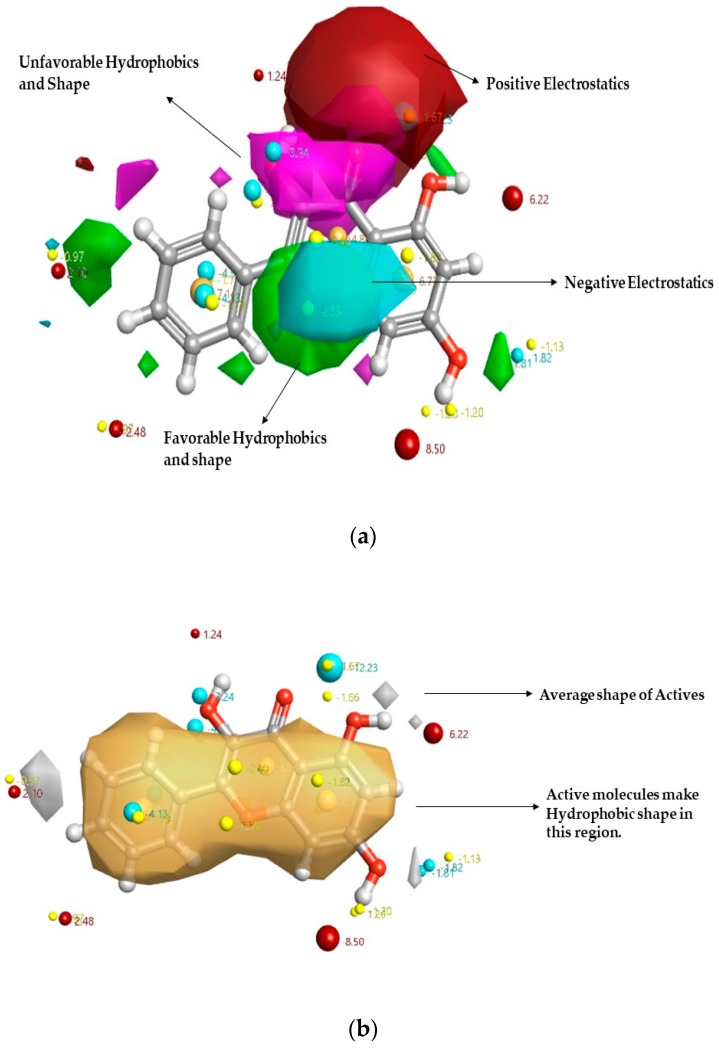
Qualitative structure–activity relationship (SAR) model of the DPP-4 inhibitor model map is superposed to galangin. The molecular insight of SAR mechanism models, revealing DPP-4 inhibitors of natural compounds, as detected through an average of actives and activity cliff summary study: (**a**) Positive field region is shown in red controls the DPP-4 inhibitory activity; green color shows favorable hydrophobic feature and pink color shows unfavorable hydrophobic shape; While the negative region in cyan: (**b**) The white region shows the inhibitory activity shape feature, and yellow color shows the hydrophobic shape of inhibitory activity.

In previous work, the Activity Atlas model was studied by Kalhotra et al. to depict the key characteristics of DPP-4 inhibition by natural compounds [31]. Figure 2 reveals the structure–activity relationship (SAR) model of DPP-4 inhibitors of natural origin, where the flavanol galangin is superposed. It was seen that galangin fits into the Activity Atlas Model built and provides the molecular insights into the inhibitory activity. The hydroxy group in the 5,7-hydroxyl group increased the electronegative and electropositive features. Hence, it was predicted that galangin would have more activity to inhibit DPP-4 than chrysin. Also, the field template tool was used to calculate the novelty score of galangin to be high or very high. It was observed that the novelty score of galangin was very high, showing that the study of galangin behavior to inhibit DPP-4 would add new information in understanding the DPP-4 inhibitor space.

**Figure 3:**
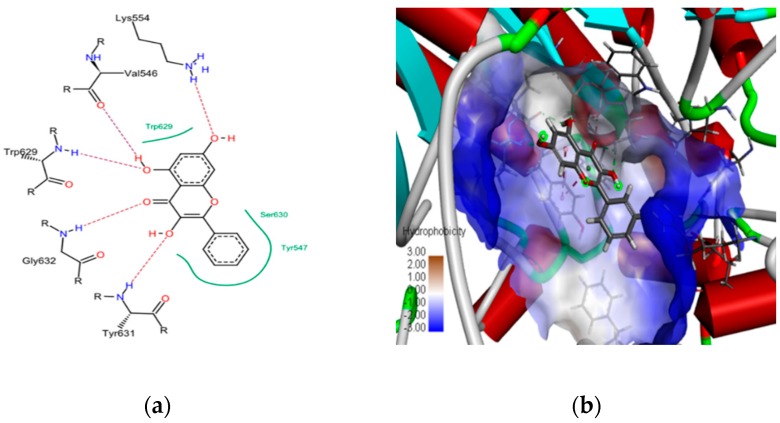
A two-dimensional view of galangin interacting residues with DPP-4 (**a**) and (**b**) the three-dimensional view showing the binding pose of galanin with DPP-4 enzyme, where hydrophobic surface features are visualized using discovery studio visualizer software.

To demonstrate that galangin is more effective than chrysin in inhibiting DPP-4, in silico molecular docking simulations were performed using the molecular docking FlexX tool, provided by Lead IT software. Galangin was docked to the active site of the DPP-4 enzyme, which resulted in ten top-scoring binding poses of galangin. The best top-ranking binding pose was chosen based on the FlexX score, and the same was visualized using Discovery Studio visualizer, as shown in Figure 3. The top-scoring pose of galangin possessed a binding energy of −24.162 kJ/mol and chrysin possessed a binding energy of −21.4 kJ/mol (potential binding sites of top 10 docking poses are shown in Figure S1). Significant interactions could be found between galangin, and the interacting residues are: LYS 554, TYR 547, ASP 545, VAL 546, LY 632, TYR 631, SER 630, GLY 633, GLY 628. The interacting residues SER 630 and TYR 547 belong to the DPP-4 druggable region. Further electrostatic interactions between galangin and the DPP-4 protein revealed the critical amino acid residues: TYR 631, GLY 632, TRP 629, VAL 546 and LYS 554, which formed hydrogen bonding interactions. TYR 457 formed Pi-Pi stacked interactions and the positively charged interaction with LYS 554 was responsible for stabilized interactions (electrostatic potential features surfaces between galangin and DPP-4 are shown in Figure S2).

### 2.2. In Vitro Studies on Validation of as Galangin Inhibiting DPP-4 Enzyme

**Figure 4:**
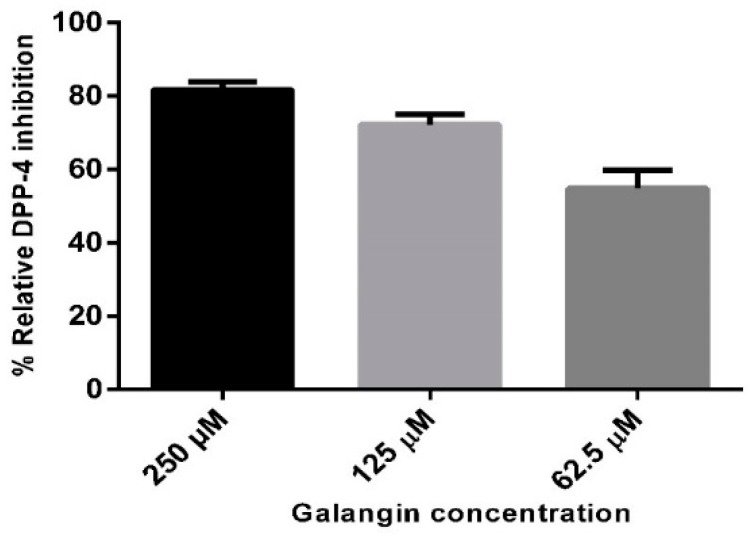
Relative percent of inhibition of DPP-4 enzyme by galangin at concentrations 250, 125 and 62.5 µM. Data are represented as mean ± standard deviation.

Three different concentrations of 250, 125, and 62.5 µM of galangin was utilized to test the DPP-4 inhibitory activity. The result showed that galangin possessed an inhibitory effect in a concentration-dependent manner. The relative percent of inhibition was 81.8%, 72.2%, and 54.93%, respectively, which is shown in Figure 4. Percent of inhibition curve calculations revealed that galangin had an IC50 value of 40.13 µM (shown in Figure S3). In our earlier work, chrysin displayed the relative percent of inhibition of 68.9% (250 µM), 38.4% (125 µM) and 16.6% (62.5 µM). Hence, the results revealed that presence of the hydroxyl group in galangin, in comparison to chrysin, had increased the inhibitory effect by 18.7% (250 µM), 33.8% (125 µM) and 23% (62.5 µM). The differences in inhibition were due to the presence of the hydroxyl group in the third position of the flavonoid galangin, in comparison to chrysin, and field point visualizations were used to study the hydrophobic, positive and negative electrostatic features (shown in Figure S4). The obtained results are the first evidence of galangin inhibiting the DPP-4 enzyme, and that the observed behavior is concentration dependent. 

Recently, the DPP-4 inhibitor anagliptin has been proven to promote skeletal muscle glucose uptake [33], and this study leads us to hypothesize that galangin can also promote skeletal muscle glucose uptake, as well the potential to promote skeletal muscle cell proliferation. Hence, we studied the acute toxicity effect of galangin on skeletal muscle cell. Successful clinical trials on sitagliptin in combination with insulin or with metformin [34,35], in reducing blood glucose levels, led us to test if the combination of galangin and insulin could be beneficial to skeletal muscle cell health. Hence, the acute toxicity effect of the galangin and insulin combination was validated in rat L6 skeletal muscle cell model.

### 2.3. Galangin in Combination with Insulin Promotes Differentiated Skeletal Muscle Cell Proliferation

**Figure 5:**
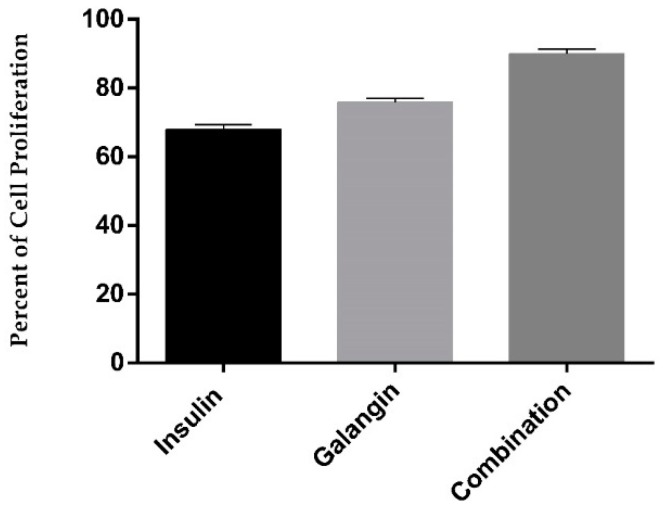
Effect on the percent of cell proliferation on differentiated rat L6 skeletal muscle cells over 24 h with different treatments of 10 nM insulin alone, 270.2 µg/mL galangin alone, and in combination (10 nM insulin and 270.2 µg/mL galangin). All data are represented in the graph as the mean ± standard deviation of six different experiments.

In the present study, the sulforhodamine B (SRB) assay was used to demonstrate the acute cell toxicity effect of treatments of 10 nM insulin, 270.2 µg/mL galangin, and the combination (10 nM insulin and 270.2 µg/mL galangin) on rat L6 skeletal muscle cells. Figure 5 shows the percent of cell proliferation, where it was observed that galangin alone and in combination effectively proliferates differentiated skeletal muscle cells significantly in comparison to insulin-treated cells (*p* < 0.05). It was observed that 270.2 µg/mL galangin, and the combination (10 nM insulin and 270.2 µg/mL galangin) was not acutely toxic to cells and was safe. 

### 2.4. Glucose Metabolism

**Figure 6:**
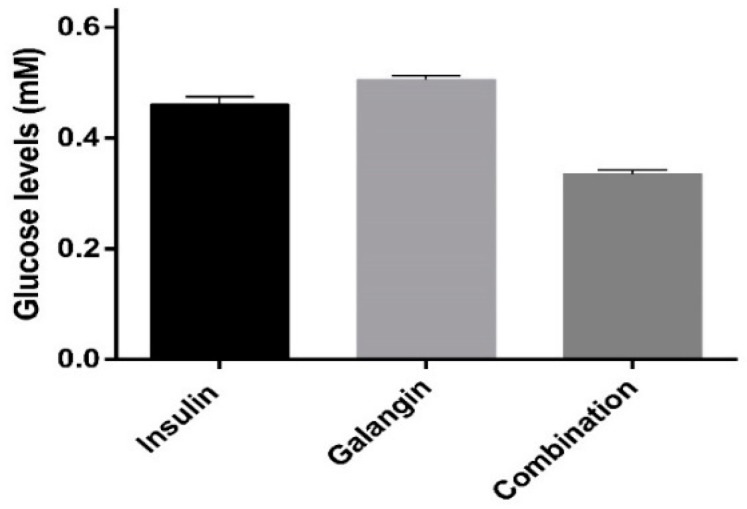
The amount of glucose levels in cell supernatant of rat L6 skeletal muscles treated with different treatments: L6 cell line (control), insulin (10 nM), galangin (250 μg/mL), and in combination (10 nM insulin and 250 μg/mL galangin) for 24 h. All the data are represented as the mean ± standard deviation in triplicate.

The glucose levels in the cell medium over different treatments—control cells (L6 cell line) with: 10 nM insulin; 270.2 µg/mL galangin; and in combination (10 nM insulin with 270.2 µg/mL galangin)—are shown in Figure 6. It was observed that the glucose levels in the combination treatment, 10 nM insulin and 270.2 µg/mL galangin treated cells were statistically significant (*p* < 0.05). It was also observed that treatment of galangin alone and in combination with insulin significantly reduced glucose levels in comparison to insulin alone (*p* < 0.05). The reduction in the glucose levels in skeletal muscle cell means the treatments can have a remarkable effect on plasma glucose level, as skeletal muscle cells represent the most substantial proportion of the human body. Amal et al. reported that galangin at different doses reduces glucose in the blood when administered orally in the streptozotocin treated diabetic model. The results of the present work can be correlated to Amal et al. to explain the possible mechanism of action of galangin in reducing glucose in the blood. In this work, it was proven that galangin inhibits DPP-4 and regulates glucose metabolism in the skeletal muscle cell line. Hence, galangin reduces blood glucose levels in the murine model [36,37]. 

## 3. Materials and Methods 

### 3.1. Chemicals and Reagents

Galangin, the human dipeptidyl peptidase-4 (DPP-4) enzyme inhibitory screening kit, dimethyl sulfoxide, sulforhodamine B (SRB), 96-well microtiter plates (Costar), and deuterium oxide (0.05 wt.% 3-(trimethylsilyl) propionic-2,2,3,3-d4 acid (TSP), sodium salt) were purchased from Sigma (St. Louis, MO, USA). Regular human insulin was obtained from AMSA Laboratories (Mexico City, Mexico). The L6 cell line (ATCC^®^ CRL-1458™), horse serum (ATCC^®^ 30-2041™), Eagle’s minimum essential medium (EMEM) with sterile-filtered L-glutamine (ATCC^®^ 30-2003™) were purchased from ATCC Global Bioresource Center (Manassas, VA, USA).

### 3.2. Molecular Docking Simulations

The three-dimensional structure of DPP-4 in complex with vildagliptin was downloaded from the Protein Data Bank, whose PDB ID is 6B1E [38]. Initially, the quality of the model was validated using the structure validation server SAVES. Then the complex was used to validate the docking protocol to study the galangin and DPP-4 protein interactions. Initially, the DPP-4 with vildagliptin complex was loaded into the protein preparation module provided by Biosolve IT software (GmnH, St Augustin, Germany). The binding site in chain A of the DPP-4 with vildagliptin complex was chosen to perform protein minimization until the atomic coordinates converged, before the molecular docking protocol was initiated. The Biosolve IT software provided the molecular docking platform, using Lead IT modules, which uses the FlexX algorithm to score, rank, and generate binding poses of galangin interaction with DPP-4 enzyme. It is well known that the FlexX algorithm utilizes an incremental construction algorithm [39]. All the docking poses, 2D and 3D protein-ligand interactions were visualized and studied using the Discovery Studio Visualizer 2.5.5.

### 3.3. In Vitro DPP-4 Inhibition Assay

Galangin inhibition of the DPP-4 enzyme was demonstrated using the DPP-4 Inhibitor screening kit (Sigma Aldrich, St. Louis, MO, USA). In the present work, the in vitro assay was carried out as per the kit instructions. Briefly, the recombinant human DPP-4 enzyme cleaves the non-fluorescent substrate, H-Gly-Pro-AMC, and releases the fluorescent product, 7-amino-4-methyl coumarin (AMC). The fluorescence emissions ((λex = 360 nm and λem = 460 nm) were collected in kinetic mode for 30 min on black clear-bottomed 96-well plates, on a microwell plate reader, the microwell plate reader FlexStation 3 Multi-Mode Microplate Reader, Molecular Devices, LLC, San Jose, CA, USA. Formula (1) was used to calculate the percent of relative inhibition, where ΔF/ΔT represented the change in fluorescence concerning the chosen time interval. GraphPad was utilized to plot the graphs obtained from all the triplicate experiments.

(1) $$\%\;\mathrm{Relative}\;\mathrm{Inhibition}=\frac{\left(\frac{∆F}{∆T}\right)\mathrm{Enzyme}-\left(\frac{∆F}{∆T}\right)\mathrm{Enzyme}\;\mathrm{Inhibitor}\;\mathrm{complex}}{\frac{∆F}{∆T}\mathrm{Enzyme}}*100$$

### 3.4. Cell Culture

The cell culture protocol described by Norio et al. [40] was utilized in this study, with slight modifications. In brief, L6 cells were cultured in EMEM medium with sterile filtered l-glutamine, 100 units/mL penicillin and 100 μg/mL streptomycin, and incubated at 37 °C in a humidified incubator with 5% CO_2_. Cells were seeded in 96-well plates at a cell density of 3 × 10^3^ cells/well, and EMEM media containing l-glutamine supplemented with 2% horse serum was used to culture the cells for 48 h, until the cells reached semiconfluency. L6 cells were differentiated for five days to form myotubes, which were then used for the cell proliferation assay.

### 3.5. Cell Proliferation Assay

Sulforhodamine B (SRB) dye was the protein binding dye used in this study to determine cell proliferation. Briefly, the differentiated L6 cells were exposed to different treatments for 24 h, fixed with cold 50% (*wt*/*vol*) TCA and stained with the protein binding dye 0.4% SRB. This was followed by washing with 1% acetic acid to remove unbound dye and to solubilize the bound dye by adding 10 mM unbuffered tris base (100 μL). The absorbance of the treatments was read at 515 nm by using the BioteK microplate reader and all the experiments are carried out in triplicate. The percent of cell proliferation was calculated using Equation (2).

(2) $$\%\;\mathrm{cell}\;\mathrm{proliferation}=\frac{\mathrm{Absorbance}\;\mathrm{of}\;\mathrm{the}\;\mathrm{sample}\;\mathrm{at}\;515\;\mathrm{nm}\;}{\mathrm{Absorbance}\;\mathrm{of}\;\mathrm{the}\;\mathrm{untreated}\;\mathrm{sample}\;\mathrm{at}\;515\;\mathrm{nm}}*100$$

### 3.6. Glucose Quantification in Cell Supernatant Using 1H-NMR Spectroscopy

In this study, the metabolite glucose was quantified as per the protocol described by Chittepu et al. [41] with slight modifications. In brief, differentiated L6 skeletal muscle cells were treated with the treatments: 10 nM insulin, 270.2 μg/mL galangin, combination (10 nM insulin with 270.2 μg/mL of galangin) for 24 h. Then, the cell supernatant was collected and stored at −20 °C for identification and quantification of glucose using 1H-NMR spectroscopy. The cell supernatant was centrifuged at 10,000 rpm for 20 min at room temperature. To the resulting 300 µL of cell supernatant, 300 µL of internal standard (deuterium oxide with 0.29 mM 3-(trimethylsilyl) propionic-2,2,3,3-d4 acid (TSP)) was added and used without further pH correction. Immediately, the resultant 600 µL supernatant was transferred to 5 mm NMR tubes for NMR analysis. A Bruker Avance 750 MHz spectrometer equipped with 5 mm TXI probe at 298 K, was used for 1H-NMR on all samples. The 1D-1H NMR spectra were acquired with 64 scans and the resulting Free Induction Decay (FID) spectra were subjected phase correction, baseline correction and chemical shift, referenced to the internal standard TSP. The Chenomx NMR Suite Program 8.2 (Chenomx Inc., Edmonton, AB, Canada) software was used to identify and quantify the targeted metabolite glucose.

### 3.7. Statistical Analysis

Graphically, all data presented were expressed as means ± standard deviation. An ordinary one-way analysis of variance (ANOVA) was used to the calculated *p*-value using Graph Pad software, and then groups with a value *p* < 0.05 were considered as statistically significant.

## 4. Conclusions

The present study demonstrates that galangin inhibits DPP-4 in vitro significantly, in comparison to chrysin, because of the presence of the hydroxyl group in the third positions of 5,7-hydroxyflavone. These findings are significant, since these are the first evidence of galangin inhibiting the human dipeptidyl peptidase-4 enzyme. The results of the treatments of 250 µg/mL galangin, and the combination (10 nM insulin-250 µg/mL of galangin) showed safety with rat L6 skeletal muscle cells and regulation of glucose metabolism significantly, in comparison to 10 nM insulin-treated skeletal muscle cells. Our findings could lead to the proof of concept to develop combination therapy (insulin and galangin) for the potential treatment of diabetes mellitus and as well, improve skeletal muscle cell health. In the future, more studies should be performed to investigate the potential of insulin and galangin to be used to prevent and manage diabetes mellitus.

## Supplementary Materials

Supplementary materials can be found at https://www.mdpi.com/1422-0067/20/5/1228/s1.

## References

[1] Ogurtsova K., da Rocha Fernandes J.D., Huang Y., Linnenkamp U., Guariguata L., Cho N.H., Cavan D., Shaw J.E., Makaroff L.E. (2017). IDF Diabetes Atlas: Global estimates for the prevalence of diabetes for 2015 and 2040. Diabetes Res. Clin. Pract..

[2] American Diabetes Association (2018). 2. Classification and Diagnosis of Diabetes: Standards of Medical Care in Diabetes—2018. Diabetes Care.

[3] La Greca A.M., MacKey E.R. (2009). Type 1 diabetes mellitus. Behavioral Approaches to Chronic Disease in Adolescence: A Guide to Integrative Care.

[4] Chaudhury A., Duvoor C., Reddy Dendi V.S., Kraleti S., Chada A., Ravilla R., Marco A., Shekhawat N.S., Montales M.T., Kuriakose K. (2017). Clinical Review of Antidiabetic Drugs: Implications for Type 2 Diabetes Mellitus Management. Front. Endocrinol..

[5] Alhadramy M.S. (2016). Diabetes and oral therapies a review of oral therapies for diabetes mellitus. J. Taibah Univ. Med. Sci..

[6] Milder T., Stocker S., Abdel Shaheed C., McGrath-Cadell L., Samocha-Bonet D., Greenfield J., Day R. (2019). Combination Therapy with an SGLT2 Inhibitor as Initial Treatment for Type 2 Diabetes: A Systematic Review and Meta-Analysis. J. Clin. Med..

[7] Moon M.K., Hur K.Y., Ko S.H., Park S.O., Lee B.W., Kim J.H., Rhee S.Y., Kim H.J., Choi K.M., Kim N.H. (2017). Combination therapy of oral hypoglycemic agents in patients with type 2 diabetes mellitus. Korean J. Intern. Med..

[8] Massi-Benedetti M., Orsini-Federici M. (2008). Treatment of type 2 diabetes with combined therapy: What are the pros and cons?. Diabetes Care.

[9] Rivas D.A., Fielding R.A. (2012). Skeletal Muscle. Encyclopedia of Human Nutrition.

[10] Lee M.J., Kim E.H., Bae S.J., Choe J., Jung C.H., Lee W.J., Kim H.K. (2018). Protective role of skeletal muscle mass against progression from metabolically healthy to unhealthy phenotype. Clin. Endocrinol..

[11] Malin S.K., Huang H., Mulya A., Kashyap S.R., Kirwan J.P. (2013). Lower dipeptidyl peptidase-4 following exercise training plus weight loss is related to increased insulin sensitivity in adults with metabolic syndrome. Peptides.

[12] Röhrborn D., Wronkowitz N., Eckel J. (2015). DPP4 in diabetes. Front. Immunol..

[13] Monami M., Iacomelli I., Marchionni N., Mannucci E. (2010). Dipeptydil peptidase-4 inhibitors in type 2 diabetes: A meta-analysis of randomized clinical trials. Nutr. Metab. Cardiovasc. Dis..

[14] Todd J.F., Bloom S.R. (2007). Incretins and other peptides in the treatment of diabetes. Diabet. Med..

[15] Liu Y., Hu Y., Liu T. (2012). Recent Advances in Non-Peptidomimetic Dipeptidyl Peptidase 4 Inhibitors: Medicinal Chemistry and Preclinical Aspects. Curr. Med. Chem..

[16] Zettl H., Schubert-Zsilavecz M., Steinhilber D. (2010). Medicinal chemistry of incretin mimetics and DPP-4 inhibitors. ChemMedChem.

[17] Kuhn B., Hennig M., Mattei P. (2007). Molecular Recognition of Ligands in Dipeptidyl Peptidase IV. Curr. Med. Chem..

[18] Pissurlenkar R.R.S., Shaikh M.S., Coutinho E.C. (2007). 3D-QSAR studies of Dipeptidyl peptidase IV inhibitors using a docking based alignment. J. Mol. Model..

[19] Meduru H., Wang Y.T., Tsai J.J.P., Chen Y.C. (2016). Finding a potential dipeptidyl peptidase-4 (DPP-4) inhibitor for type-2 diabetes treatment based on molecular docking, pharmacophore generation, and molecular dynamics simulation. Int. J. Mol. Sci..

[20] Lovshin J.A., Drucker D.J. (2009). Incretin-based therapies for type 2 diabetes mellitus. Nat. Rev. Endocrinol..

[21] Thomsen R.W., Pedersen L., Møller N., Kahlert J., Beck-Nielsen H., Sørensen H.T. (2015). Incretin-based therapy and risk of acute pancreatitis: A nationwide population-based case-control study. Diabetes Care.

[22] Cragg G.M., Newman D.J. (2013). Natural products: A continuing source of novel drug leads. Biochim. Biophys. Acta-Gen. Subj..

[23] Cragg G.M., Newman D.J., Snader K.M. (1997). Natural products in drug discovery and development. J. Nat. Prod..

[24] Zhang L., Demain A.L. (2005). Natural Products: Drug Discovery and Therapeutic Medicine.

[25] Rienks J., Barbaresko J., Oluwagbemigun K., Schmid M., Nöthlings U. (2018). Polyphenol exposure and risk of type 2 diabetes: Dose-response meta-analyses and systematic review of prospective cohort studies. Am. J. Clin. Nutr..

[26] Unnikrishnan M.K., Veerapur V., Nayak Y., Mudgal P.P., Mathew G. (2013). Antidiabetic, Antihyperlipidemic and Antioxidant Effects of the Flavonoids. Polyphenols in Human Health and Disease.

[27] Fantini M., Benvenuto M., Masuelli L., Frajese G.V., Tresoldi I., Modesti A., Bei R. (2015). In vitro and in vivo antitumoral effects of combinations of polyphenols, or polyphenols and anticancer drugs: Perspectives on cancer treatment. Int. J. Mol. Sci..

[28] Kim Y., Narayanan S., Chang K.O. (2010). Inhibition of influenza virus replication by plant-derived isoquercetin. Antivir. Res..

[29] Daglia M. (2012). Polyphenols as antimicrobial agents. Curr. Opin. Biotechnol..

[30] Khurana S., Venkataraman K., Hollingsworth A., Piche M., Tai T.C. (2013). Polyphenols: Benefits to the cardiovascular system in health and in aging. Nutrients.

[31] Pérez-Durillo F., Segarra A., Villarejo A., Ramírez-Sánchez M., Prieto I. (2018). Influence of Diet and Gender on Plasma DPP4 Activity and GLP-1 in Patients with Metabolic Syndrome: An Experimental Pilot Study. Molecules.

[32] Kalhotra P., Chittepu V., Osorio-Revilla G., Gallardo-Velázquez T. (2018). Structure-Activity Relationship and Molecular Docking of Natural Product Library Reveal Chrysin as a Novel Dipeptidyl Peptidase-4 (DPP-4) Inhibitor: An Integrated In Silico and In Vitro Study. Molecules.

[33] Sato H., Kubota N., Kubota T., Takamoto I., Iwayama K., Tokuyama K., Moroi M., Sugi K., Nakaya K., Goto M. (2016). Anagliptin increases insulin-induced skeletal muscle glucose uptake via an NO-dependent mechanism in mice. Diabetologia.

[34] Kutoh E. (2011). Sitagliptin is effective and safe as add-on to insulin in patients with absolute insulin deficiency: A case series. J. Med. Case Rep..

[35] Shankar R.R., Bao Y., Han P., Hu J., Ma J., Peng Y., Wu F., Xu L., Engel S.S., Jia W. (2017). Sitagliptin added to stable insulin therapy with or without metformin in Chinese patients with type 2 diabetes. J. Diabetes Investig..

[36] Aloud A.A., Chinnadurai V., Govindasamy C., Alsaif M.A., Al-Numair K.S. (2018). Galangin, a dietary flavonoid, ameliorates hyperglycaemia and lipid abnormalities in rats with streptozotocin-induced hyperglycaemia. Pharm. Biol..

[37] Aloud A.A., Veeramani C., Govindasamy C., Alsaif M.A., El Newehy A.S., Al-Numair K.S. (2017). Galangin, a dietary flavonoid, improves antioxidant status and reduces hyperglycemia-mediated oxidative stress in streptozotocin-induced diabetic rats. Redox Rep..

[38] Berman H.M., Battistuz T., Bhat T.N., Bluhm W.F., Bourne P.E., Burkhardt K., Feng Z., Gilliland G.L., Iype L., Jain S. (2002). The protein data bank. Acta Crystallogr. Sect. D Biol. Crystallogr..

[39] Kramer B., Rarey M., Lengauer T. (1999). Evaluation of the FlexX incremental construction algorithm for protein-ligand docking. Proteins Struct. Funct. Genet..

[40] Yamamoto N., Sato T., Kawasaki K., Murosaki S., Yamamoto Y. (2006). A nonradioisotope, enzymatic assay for 2-deoxyglucose uptake in L6 skeletal muscle cells cultured in a 96-well microplate. Anal. Biochem..

[41] Chittepu V., Kalhotra P., Gallardo-Velázquez T., Robles-de la Torre R., Osorio-Revilla G., Chittepu V.C.S.R., Kalhotra P., Gallardo-Velázquez T., Robles-de la Torre R.R., Osorio-Revilla G. (2018). Designed Functional Dispersion for Insulin Protection from Pepsin Degradation and Skeletal Muscle Cell Proliferation: In Silico and In Vitro Study. Nanomaterials.

