# Supplementary material for: Discovery of Galangin as a Potential DPP-4 Inhibitor That Improves Insulin-Stimulated Skeletal Muscle Glucose Uptake: A Combinational Therapy for Diabetes
Source: Int J Mol Sci. 2019 Mar 11;20(5):1228. doi: 10.3390/ijms20051228 (PMC6429117; doi:10.3390/ijms20051228)
Supplement: Supplementary file 1 [file ijms-20-01228-s001.pdf]

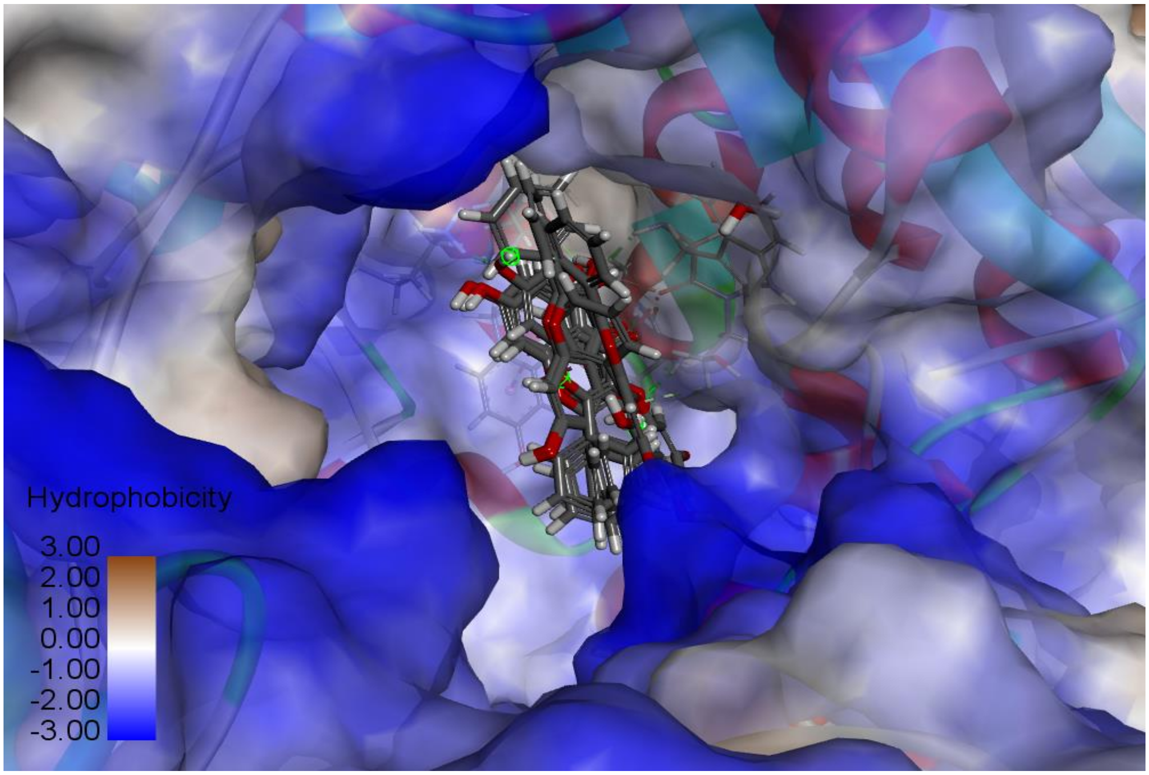

Figure S1. Top 10 docking poses of natural flavonoid Galangin at the druggable region of DPP4.

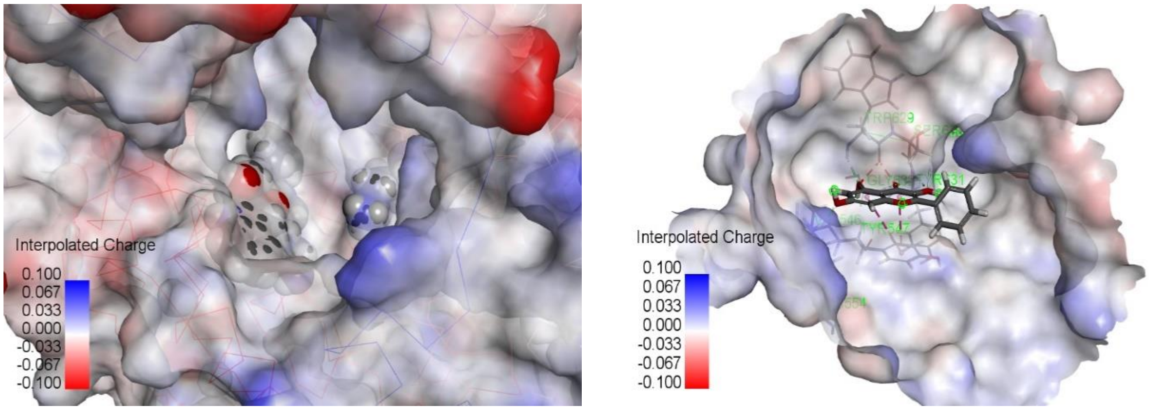

(a) **(b)**

Figure S2. (a) The electrostatic charge surface of druggable region DPP4 glycoprotein and (b) galangin binding at the druggable region of DPP4. Blue color denotes positively charged and Red color denotes negatively charged.

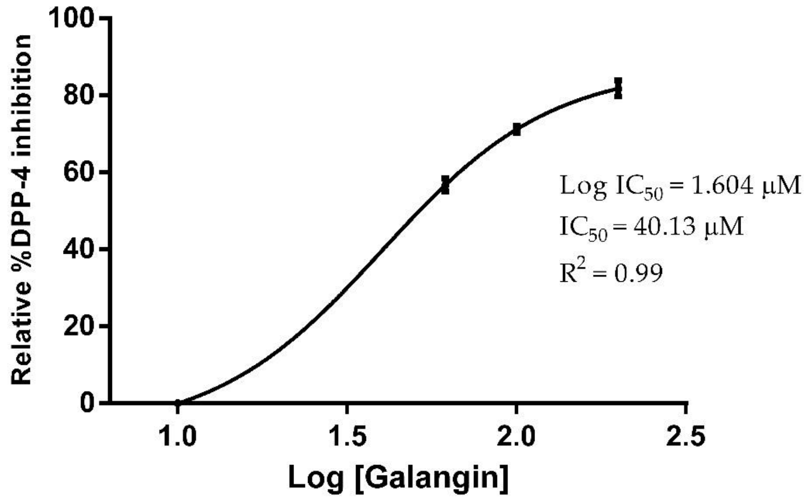

Figure S3. The dose-response curve of galangin inhibiting DPP4 at three different concentration.

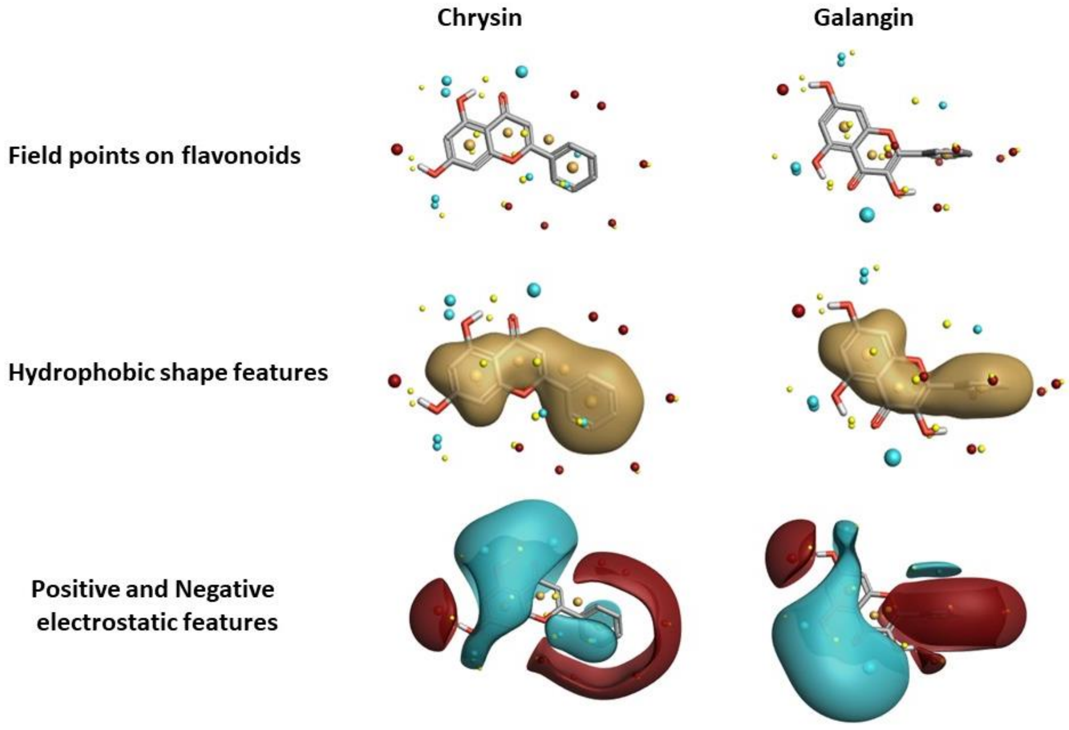

Figure S4. Molecular insights of Chrysin and Galangin calculated using activity cliff summary.
